# Cathodoluminescence spectroscopy of monolayer hexagonal boron nitride

**DOI:** 10.1038/s41598-023-50502-9

**Published:** 2024-01-02

**Authors:** Kohei Shima, Tin S. Cheng, Christopher J. Mellor, Peter H. Beton, Christine Elias, Pierre Valvin, Bernard Gil, Guillaume Cassabois, Sergei V. Novikov, Shigefusa F. Chichibu

**Affiliations:** 1 Institute of Multidisciplinary Research for Advanced Materials, Tohoku University, Sendai, 980-8577 Japan; 2 School of Physics and Astronomy, University of Nottingham, Nottingham, NG7 2RD UK; 3 Laboratoire Charles Coulomb, UMR5221 CNRS, Université de Montpellier, 34095 Montpellier, France

**Keywords:** Two-dimensional materials, Two-dimensional materials

## Abstract

Cathodoluminescence (CL) spectroscopy is a suitable technique for studying the luminescent properties of optoelectronic materials because CL has no limitation on the excitable bandgap energy and eliminates ambiguous signals due to simple light scattering and resonant Raman scattering potentially involved in the photoluminescence spectra. However, direct CL measurements of atomically thin two-dimensional materials have been difficult due to the small excitation volume that interacts with high-energy electron beams. Herein, distinct CL signals from a monolayer hexagonal BN (hBN), namely mBN, epitaxial film grown on a graphite substrate are shown by using a CL system capable of large-area and surface-sensitive excitation. Spatially resolved CL spectra at 13 K exhibited a predominant 5.5-eV emission band, which has been ascribed to originate from multilayered aggregates of hBN, markedly at thicker areas formed on the step edges of the substrate. Conversely, a faint peak at 6.04 ± 0.01 eV was routinely observed from atomically flat areas, which is assigned as being due to the recombination of phonon-assisted direct excitons of mBN. The CL results support the transition from indirect bandgap in bulk hBN to direct bandgap in mBN. The results also encourage one to elucidate emission properties of other low-dimensional materials by using the present CL configuration.

## Introduction

Two-dimensional (2D) layered materials, such as graphite, hexagonal boron nitride (hBN), and transition metal dichalcogenides (TMDs), are the building blocks of van der Waals (vdW) heterostructures^1,2^ that are promising platform for optoelectronics^3^ and valleytronics^4,5^. An isolation of monolayer 2D materials causes plenty of optoelectrical phenomena. For example, Mak et al.^3^ have concluded a transition from indirect bandgap in bulk molybdenum disulfide (MoS_2_) to direct bandgap in monolayer MoS_2_, because the monolayer MoS_2_ exhibited an increased luminescence quantum efficiency by more than four orders of magnitude compared with the bulk MoS_2_^3^. For realizing vdW heterostructures consisting of different 2D materials with desired band alignment and interlayer coupling, it is essential to understand the fundamental optoelectronic properties of 2D materials.

hBN crystallizes in layers of a 2D honeycomb structure based on in-plane three *sp*^2^ covalent bonds that are connected by out-of-plane π bonds. Accordingly, hBN is a key building block in vdW heterostructures based on graphite^2^, because of the large bandgap energy (${E}_{{\text{g}}}$) of 6 eV^6,7^ and a small lattice mismatch (~ 1.8%) to graphite. On another front, hBN is an exotic candidate for the use in deep ultraviolet (DUV) light emitters despite its indirect bandgap^6^: a lasing action at 5.77 eV has been reported by Watanabe et al.^8^ from hBN single crystals grown by the high-pressure high-temperature synthesis, followed by the operation of a planar DUV light-emitting device^9^. With respect to condensed matter physics of hBN, Cassabois et al.^6^ have revealed that hBN has an indirect bandgap with the nonphonon (NP) indirect exciton (iX) energy of 5.955 eV at 10 K: iXs built from **M** and **K** points of the Brillouin zone (BZ) for the conduction and valence bands, respectively^10–13^, require the scattering by phonons^14,15^ of wavevector **MK** to fulfill momentum conservation during photon absorption or emission in bulk hBN. Nevertheless, the near-band-edge (NBE) emission of hBN, namely LO(T) and TO(T) phonon-assisted iXs [iX_LO(T)_ and iX_TO(T)_, respectively]^6^, where T indicates **T** point of the BZ, exhibited markedly high internal quantum efficiency of 50% at 10 K^7^. Such a high internal quantum efficiency has been ascribed to originate from macroscopic degeneracy of the parallel transitions between the flat bands along the **KH** and **ML** lines of the Brillouin zone^16^.

In contrast to bulk hBN, very little is known about the optical transition properties of hBN of reduced layer numbers. Theoretical calculations have predicted a direct bandgap at **K** point for a monolayer hBN (mBN)^12,17^ and an indirect bandgap or marginally direct bandgap for stackings of two or more layers^17–19^, in analogy with MoS_2_^3^. Elias et al.^20^ have confirmed the presence of the direct bandgap with ${E}_{{\text{g}}}$ of 6.1 eV in the mBN epilayers grown by high-temperature plasma-assisted molecular beam epitaxy (HT-PAMBE) method on a highly oriented pyrolytic graphite (HOPG) substrate^21–23^, by means of optical reflectance (OR) and photoluminescence (PL) measurements: the PL spectrum of mBN at 10 K has consisted of doublet peaks at around 6.08 eV and 6.05 eV, which have been interpreted as the recombination of an NP direct exciton (dX), namely dX_NP_, and a ZA(K) phonon^24,25^-assisted dX [dX_ZA(K)_], respectively^20^. The 6.1-eV emission peaks associated with the recombination of dXs have been additionally verified by the optical probing techniques^26–29^.

For the accurate understanding of a luminescence spectrum of mBN, the complementary use of cathodoluminescence (CL) measurements is preferred because an electron beam (*e*-beam) excitation has no limitations on excitable ${E}_{{\text{g}}}$ and eliminates ambiguous signals due to simple light scattering and resonant Raman scattering potentially involved in PL spectra^20^. Schué et al.^30^ have carried out conventional CL measurements on exfoliated hBN flakes to record a series of CL spectra as a function of the number of hBN layers from 100 down to 6. Their CL spectra^30^ exhibited the NBE emissions at 10 K with the highest energy peak at 5.9 eV, which was followed by several phonon replicas. They^30^ also observed a thickness-dependent energy shift of the 5.9 eV peak by a few tens of meV^30^. However, CL signals of the hBN flakes thinner than 5 layers have not been shown due presumably to the small excitation volume of ultrathin hBN films including mBN and to the finite depth of the projected range of an *e*-beam in the CL system equipped on the scanning electron microscopy (SEM), which gave rise to the surface-insensitive excitation. Here we note that, for the cases of monolayer TMDs, CL signals have been recorded only when the monolayer TMDs have been encapsulated with hBN layers, where the wide *E*_g_ hBN layer functioned as an e-beam absorber for increasing the number of excited carriers that were injected in the monolayer TMDs^31–36^. However, such artificial vdW structures^31–36^ are not feasible when measuring an mBN itself.

In this paper, distinct CL signals from the mBN epilayer^21–23^ grown by HT-PAMBE on an HOPG substrate are displayed. For overcoming the drawbacks of conventional CL measurement, a home-made CL system^37^ that is capable of large-area and surface-sensitive excitation was used. The CL spectra at 13 K exhibited a predominant 5.5-eV emission band and a faint peak at 6.04 ± 0.01 eV. Since the latter energy agreed with the PL peak of 6.05 eV (10 K) that has been assigned as being due to the recombination of dX_ZA(K)_ in mBN^20^, the CL peak at 6.04 ± 0.01 eV most likely originates from mBN. The result supports the direct bandgap with the energy of 6.1 eV of mBN^20^. With respect to the 5.5-eV band, which has been ascribed to originate from multilayered aggregates of hBN^20,21,23^, spatially resolved cathodoluminescence (SRCL) measurement revealed that the emissions were localized markedly at thicker areas formed on step edges of the HOPG substrate.

## Results and discussion

**Figure 1:**
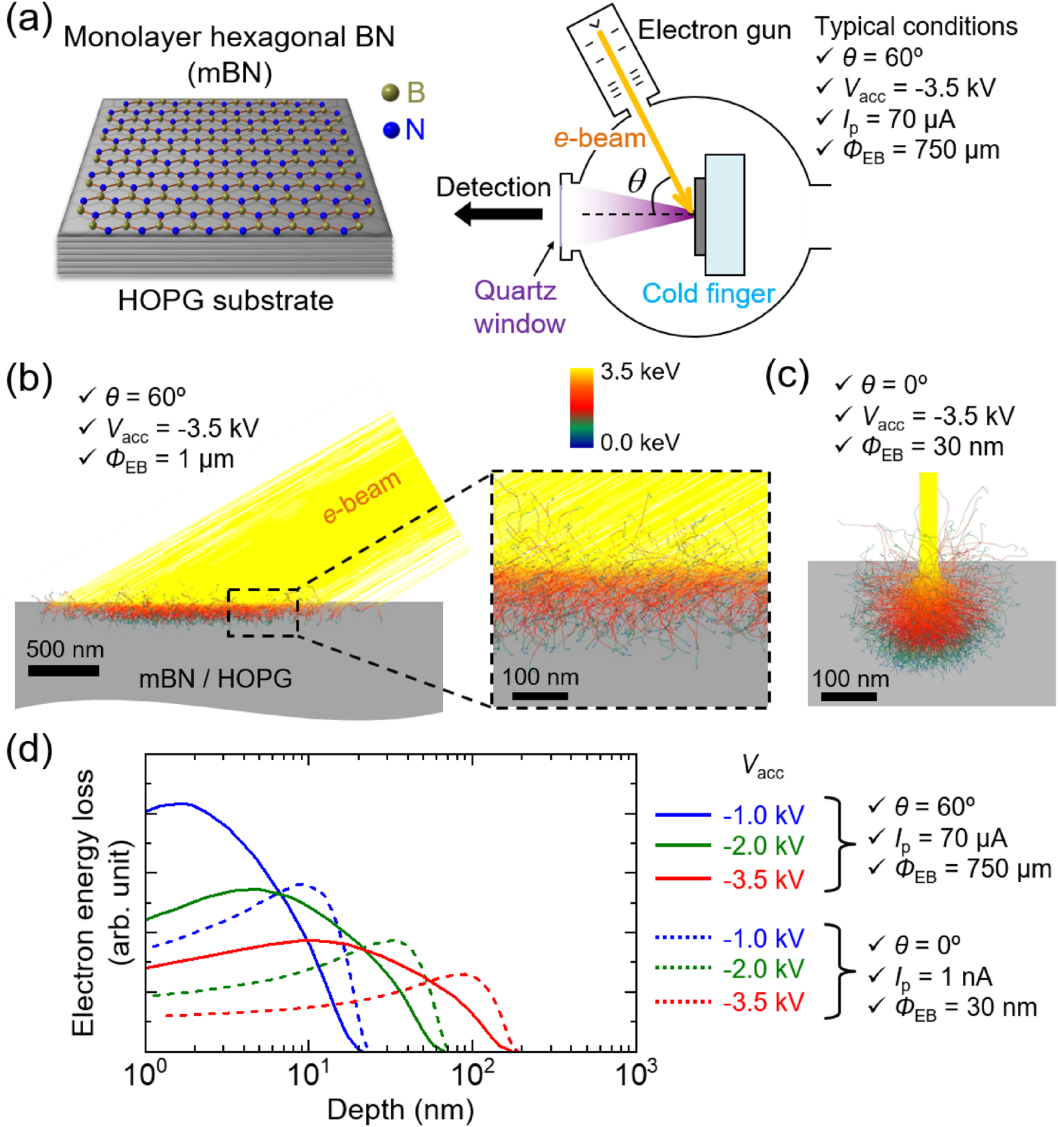
(**a**) Schematic representation of the wide-area cathodoluminescence (CL) measurement of monolayer hexagonal BN (mBN) epilayer grown on a highly oriented pyrolytic graphite (HOPG) substrate. The typical incident angle ($\theta$), acceleration voltage (${V}_{{\text{acc}}}$), probe current (${I}_{{\text{p}}}$), and diameter (${\phi }_{{\text{EB}}}$) of the *e*-beam were 60°, − 3.5 kV, 70 μA, and 750 μm, respectively. Monte Carlo simulations of the electron trajectories into a model mBN/HOPG for (**b**) the wide-area CL and (**c**) the standard spatially resolved CL (SRCL) measurements. They were calculated using the CASINO software^38^. In the simulation, $\theta$, ${V}_{{\text{acc}}}$, and ${\phi }_{{\text{EB}}}$ were set respectively to 60°, − 3.5 kV, and 1 μm for the wide-area CL and 0°, − 3.5 kV, and 30 nm for the standard SRCL measurements. (**d**) Simulated depth distributions of the total energy loss of irradiated electrons into mBN/HOPG for the wide-area CL (solid lines) and the standard SRCL (dashed lines) measurements with ${V}_{{\text{acc}}}$ = − 1.0, − 2.0, and − 3.5 kV.

Wide-area CL measurement was carried out on the mBN epilayer on an HOPG substrate at low temperatures using a homemade CL system^37^, as schematically shown in Fig. 1a. In order to increase the gross excitation volume of an ultrathin film using an *e*-beam, large-area and surface-sensitive excitation was realized by adjusting the incident angle ($\theta$), acceleration voltage (${V}_{{\text{acc}}}$), and probe current (${I}_{{\text{p}}}$) of the *e*-beam to 60°, − 3.5 kV, and 70 μA, respectively. As a result, an *e*-beam diameter (${\phi }_{{\text{EB}}}$) of approximately 750 μm and probe current density (${J}_{{\text{p}}}$) of 16 mA cm^−2^ were obtained. These parameters were varied to study the influences on the CL intensities from the mBN epilayer. Conventional SRCL measurements were carried out at low temperatures using the system equipped on the SEM (JSM-6510) under the conditions of $\theta$ = 0°, ${V}_{{\text{acc}}}$ = − 2.0 kV, and ${I}_{{\text{p}}}$ = 8 nA, giving ${\phi }_{{\text{EB}}}$ of approximately 100 nm and ${J}_{{\text{p}}}$ of 102 A cm^−2^. The dimensions of excitation volumes for the mBN/HOPG structure were calculated using a Monte Carlo simulator, CASINO software^38^. Figure 1b and c show the simulated electron trajectories into a model mBN/HOPG structure for the wide-area CL and SRCL measurements, respectively. In the simulation, $\theta$, ${V}_{{\text{acc}}}$, and ${\phi }_{{\text{EB}}}$ were set respectively to 60°, − 3.5 kV, and 1 μm for the wide-area CL and 0°, − 3.5 kV, and 30 nm for the SRCL measurements. The simulated depth distributions of the total energy loss of irradiated electrons into mBN/HOPG structure with ${V}_{{\text{acc}}}$ = − 1.0, − 2.0, and − 3.5 kV for the wide-area CL and SRCL measurements are shown by solid and dashed lines, respectively, in Fig. 1d. Compared with standard SRCL measurement, our wide-area CL appears to enable markedly surface-sensitive excitation, which contributes to increase gross CL intensity of the ultrathin mBN epilayer. While the excited carriers in the mBN epilayers were likely transferred to the conducting HOPG substrate, some carriers might remain in mBN, contributing to the radiative recombination process. The electrons irradiated with a low ${V}_{{\text{acc}}}$ were primarily stopped in the HOPG layer and scattered in the vicinity of mBN. This resulted in a gradient of carrier concentration decreasing from the center of the excitation volume toward both the surface (i.e., the mBN side) and the bulk, as illustrated in Fig. 1d. Consequently, the accumulation of carriers just below the mBN likely prolonged the residence time for some of the excited carriers within the mBN. The acquisition times were 180–300 s and 60 s for the wide-area CL and SRCL measurements, respectively. Notably, during the wide-area CL measurements, the CL intensity at 13 K decreased to approximately 70% by approximately 30 min. Meanwhile, during the SRCL measurements, the CL intensities at 10 K decreased more drastically due to the four orders of magnitude higher probe current densities. Such decrease in CL intensities likely stem from surface degradations of the mBN epilayer or the underlying HOPG induced by *e*-beam irradiations and/or burn-in of carbon (graphitic) on the surface.

**Figure 2:**
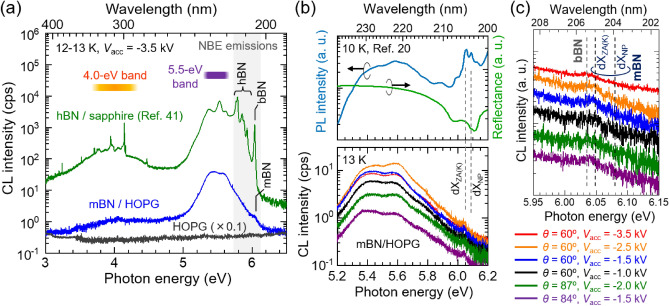
A wide-area CL spectrum at 13 K of the mBN epilayer (blue solid line). For comparison, wide-area CL spectra of a bare HOPG substrate (13 K, gray solid line) and 1-μm-thick hBN epilayers^41^ (12 K, green solid line) that was grown on a (0001) sapphire by low-pressure chemical vapor deposition using a BCl_3_–NH_3_–N_2_ gas system are also displayed. $\theta$, ${V}_{{\text{acc}}}$, ${I}_{{\text{p}}}$, ${\phi }_{{\text{EB}}}$, and probe current (${J}_{{\text{p}}}$) of the *e*-beams were 60°, − 3.5 kV, 70 μA, 750 μm, and 16 mA cm^−2^, respectively. The acquisition times were 180–300 s. NBE and bBN stand for near-band-edge and bernal BN, respectively. [Partially reproduced with permission from Ref.^41^, Appl. Phys. Lett. **120**, 231904 (2022). Copyright 2022 AIP Publishing LLC]. (**b**) The NBE CL spectra at 13 K of the mBN epilayer measured under various $\theta$ and ${V}_{{\text{acc}}}$. For reference, OR and PL spectra^20^ of the same series^20^ of mBN/HOPG measured at 10 K are displayed. [Partially reproduced with permission from Ref.^20^, Nat. Commun. **10**, 2639 (2019). Copyright 2010 Springer Nature Limited]. ${{\text{dX}}}_{{\text{NP}}}$ and ${{\text{dX}}}_{{\mathrm{ZA}}({\mathrm{K}})}$ stand for a nonphonon (NP) direct exciton (dX) and a ZA(K) phonon-assisted dX, respectively. (**c**) An enlarged view of the CL spectra between 5.95 and 6.15 eV of panel (**b**). The CL spectra are vertically offset for better visibility.

A wide-area CL spectrum at 13 K of the mBN epilayer is shown by blue solid line in Fig. 2a. For comparison, wide-area CL spectra of a bare HOPG substrate (13 K, gray solid line) and approximately 1-μm-thick hBN epilayer (12 K, green solid line) that was grown on a (0001) sapphire by low-pressure chemical vapor deposition (LP-CVD) using a BCl_3_–NH_3_–N_2_ gas system^39–41^ are also displayed. The CL spectrum of the mBN consisted of a predominant multi-peaked broad emission band at around 5.5 eV and a faint shoulder originating from certain independent emission peak at approximately 6.04 eV. Only stray light was recorded in the reference spectrum of the bare HOPG, giving a proof that the 5.5-eV band and 6.04-eV peak originate from the mBN epilayer. The 5.5-eV band^42–47^ has been found in hBN samples^41–48^ and assigned as the emissions of iX_LO(T)/TO(T)_ further scattered by multiple TO(K) phonons [iX_LO(T)/TO(T)+*n*TO(K)_, *n* is an integer]^47^ and other iXs trapped by certain stacking defects^44–47^. The 5.5-eV band has also been found in the PL spectrum at 10 K of the same series^20^ of mBN/HOPG grown by HT-PAMBE. The origin of the 5.5-eV band will be discussed later. The energy of the 6.04-eV CL peak nearly agreed with the PL peak at 6.05 eV (10 K) of the same series^20^ of mBN samples, where the PL peak has been assigned as being due to the recombination of dX_ZA(K)_ in mBN in accordance with the results of OR and PL measurements^20^. Meanwhile, the CL spectrum of the LP-CVD hBN film exhibited three emission groups labeled “NBE emissions”, “5.5-eV band”, and “4.0-eV band”^41^. Among these, the origin of the 4.0-eV band^40,42,48–54^ has been suggested to associate with carbon and oxygen impurities and a nitrogen vacancy (V_N_)^42,48,50–53^. We note that the NBE emissions of the LP-CVD hBN were accompanied by a distinct peak at around 6.035 eV at 12 K that has been detected from polytypic segments^41^, most probably graphitic bernal BN (bBN)^55–57^. As shown in Fig. 2a, the CL intensity for the NBE emission of mBN at 6.04 eV was less than three (less than four) orders of magnitude lower than that of the ZA(T) phonon-assisted iXs [iX_ZA(T)_]^6^ at 5.92 eV (iX_TO(T)_ at 5.79 eV) of the LP-CVD hBN film^39–41^. Because the thickness of mBN (i.e., 0.3–0.4 nm)^21^ was approximately 3000 times smaller than that of the LP-CVD hBN film (i.e., 1 μm)^39–41^, external quantum efficiencies for the present mBN and the LP-CVD hBN film^39–41^ appear to be the same order of magnitude.

The NBE CL spectra at 13 K of the mBN epilayer are shown in the bottom panel of Fig. 2b. For reference, the OR and PL spectra of the same series of mBN/HOPG measured at 10 K by Elias et al.^20^ are reproduced in the top panel of Fig. 2b. The values of $\theta$ and ${V}_{{\text{acc}}}$ for the CL measurement were varied to maximize overall emission intensity from the mBN epilayer. Regardless of excitation conditions, the mBN epilayer exhibited a distinct CL peak at around 6.04 eV. As described in the preceding paragraph, the peak energy nearly agreed with that of dX_ZA(K)_ of mBN (6.05 eV at 10 K)^20^. However, the other PL peak of 6.08 eV at 10 K that has been attributed to dX_NP_ of mBN by Elias et al. using PL measurements^20^, was not clearly observed in the present CL spectra, potentially due to insufficient intensity. The intensity of the CL peak at 6.04 eV exhibited a maximum under the condition of $\theta$ = 60° and ${V}_{{\text{acc}}}$ = − 2.5 kV, of which spectrum is drawn by an orange solid line in Fig. 2b. Further higher $\theta$ up to 87° resulted in lower CL intensities presumably due to the reflection of the irradiated *e*-beam at or right below the surface. Further lower ${V}_{{\text{acc}}}$ down to − 1.0 kV also resulted in lower CL intensities, because the *e*-beam was less converged and gave lower ${J}_{{\text{p}}}$.

The CL spectra between 5.95 and 6.15 eV of Fig. 2b are enlarged in Fig. 2c, where the spectra are vertically offset for better visibility. The NBE emission of the mBN epilayer was observed at 6.04 ± 0.01 eV in all the spectra. This energy range again coincide with the dX_ZA(K)_ of mBN^20^. Although the energy of the CL peak at 6.04 ± 0.01 eV is close to that of the bBN emission at 6.035 eV at 12 K^41^, such possibility is ruled out because bBN is built from at least two mBN layers with AB stacking^56^ while approximately 89% of the surface of our sample was covered by mBN. Consequently, the CL peak at 6.04 ± 0.01 eV at 13 K can be assigned as being due to the recombination of dX_ZA(K)_^20^. With respect to the insufficient intensity of the dX_NP_ peak, the result being consistent with the PL results by Elias et al.^20^ that dX_NP_ was weaker than dX_ZA(K)_, likely arose from strong exciton-phonon interaction^20,28^. Nevertheless, the present CL results strongly support the direct bandgap of 6.1 eV for mBN, which has been determined in Ref.^20^ based on the OR and PL measurements, thanks to the fact that CL is free from an excitable ${E}_{{\text{g}}}$ limits or ambiguous signals due to simple light scattering and resonant Raman scattering occasionally observed in PL spectra. The full-width of half-maximum (FWHM) of the CL peak associated with ZA phonons in thick hBN films was 20 meV at 12 K^41^. In contrast, the FWHM value was 40 meV at 13 K for the mBN epilayer in this study. The result is consistent with previously reported values of the FWHM of the PL peaks assigned as the dX_ZA(K)_ of mBN ranging from 32 to 67 meV at 10 K^20,29^. The greater FWHM value for the mBN film may originate from inhomogeneous strain distribution within the *e*-beam spot size: the relatively sharper peak observed in the hBN film compared with those in the present mBN likely originate from better spatial homogeneity of the residual strain^58–60^, because the hBN film showed a columnar structure that may suffer from weak and more homogeneous lattice stress. The intensity of the NBE emissions of mBN may decrease with temperature rise, making the CL measurements further challenging. For instance, a temperature increase from 10 to 300 K has been reported to result in quenching of the NBE emissions of mBN to approximately 30–40%, as is the case with direct bandgap semiconductors^20,29^. This is in contrast to the retrograde thermal quench behavior observed in iXs in the thick hBN films^41^.

**Figure 3:**
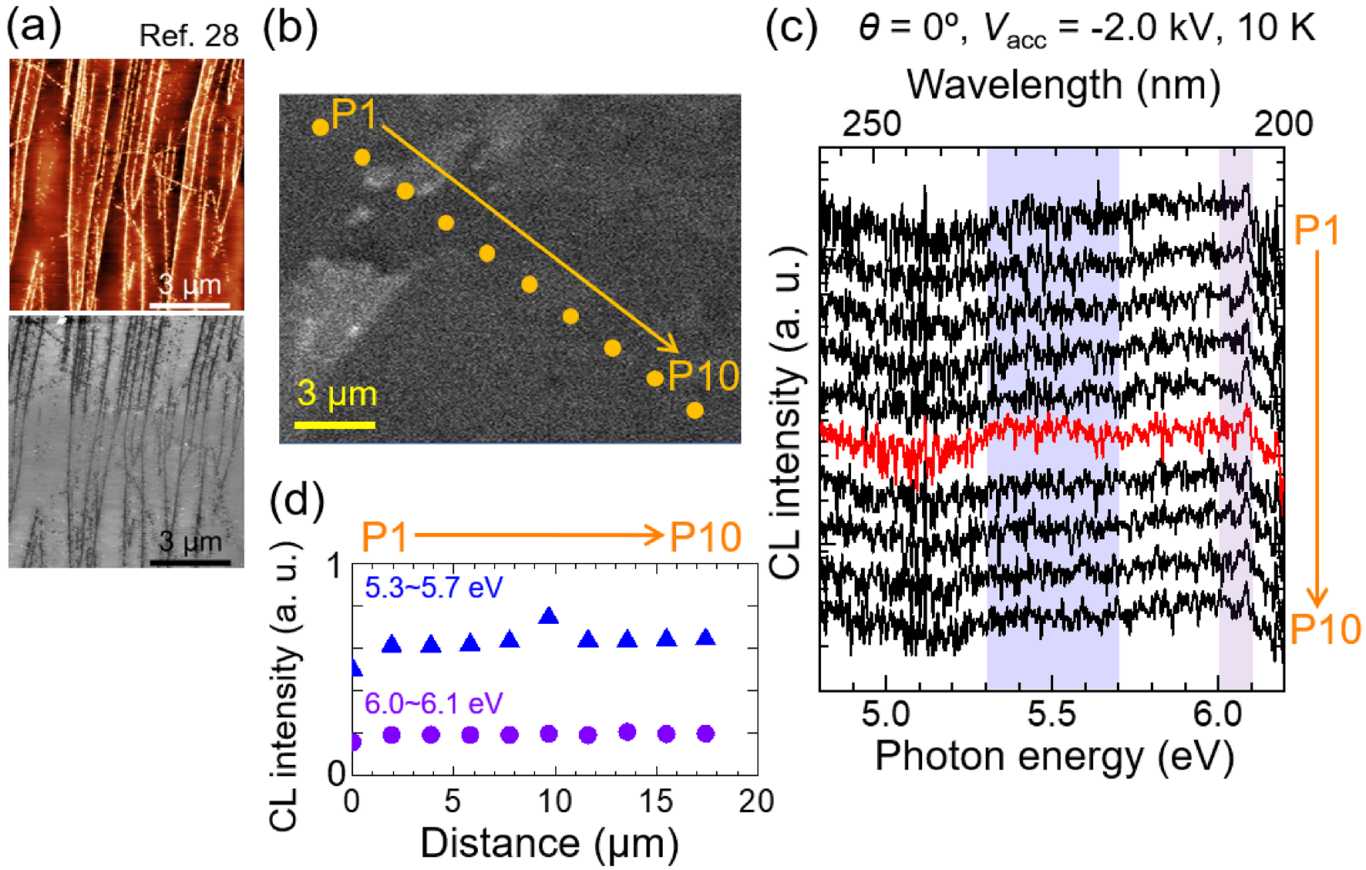
(**a**) Atomic force microscopy surface topographic (upper) and corresponding phase (bottom) images^28^, and (**b**) an SEM image of the mBN epilayer. [Partially reproduced with permission from Ref.^28^, Phys. Rev. X **12**, 011057 (2022). Copyright 2022 American Physical Society]. (**c**) Local CL spectra at 10 K measured at the positions labeled P1–P10 in panel (**b**). $\theta$, ${V}_{{\text{acc}}}$, ${I}_{{\text{p}}}$, ${\phi }_{{\text{EB}}}$, and ${J}_{{\text{p}}}$ of the *e*-beams were 0°, − 2.0 kV, 8 nA, 100 nm, and 102 A cm^−2^, respectively. The acquisition time was 60 s at each position. The CL spectra are vertically offset for better visibility. (**d**) CL intensity profiles of the NBE emission and the 5.5-eV band along the positions labeled P1–P10 in panel (**b**). The spectral integrations were carried out at the photon energies ($h\nu$) of 6.0–6.1 eV and 5.3–5.7 eV for the NBE emission and the 5.5-eV band, respectively.

The spatial distributions of CL intensities for the NBE emission and the 5.5-eV band in the mBN epilayer were studied by conventional SRCL measurement using low ${V}_{{\text{acc}}}$, as follows. One of the origins of 5.5-eV band is iXs trapped by certain stacking defects^27,44–47^. Such stacking defects can be created in multilayered aggregates of hBN formed on step edges of the HOPG substrate^20,21,23^. Figure 3a shows the surface topographic (upper) and corresponding phase (bottom) images^28^ of the same series of mBN/HOPG epilayer^20^. Brighter regions in the upper image correspond to topographically higher regions due to aggregation of BN sheets at step edges of HOPG, while white regions in the bottom image corresponded to partially exposed surface of the HOPG substrate^20,21,23^. The surface coverage of the multilayered aggregates (hBN) in the present epilayer was estimated to be approximately 11%, where the aggregates were located at every a few micrometers. In order to correlate the locations of the aggregates and local CL spectra, spot-excitation CL measurements were carried out at the positions labeled P1–P10 in the SEM image of the mBN epilayer, as shown in Fig. 3b. The values of $\theta$, ${V}_{{\text{acc}}}$, ${I}_{{\text{p}}}$, ${\phi }_{{\text{EB}}}$, and ${J}_{{\text{p}}}$ were 0°, − 2.0 kV, 8 nA, 100 nm, and 102 A cm^−2^, respectively. The local CL spectra are shown in Fig. 3c, where the spectra are vertically offset for better visibility. As shown, the local CL spectra exhibited noisy line shapes with low S/N ratios, because the emission intensities of mBN (and partial hBN) under the SRCL measurement were far lower than those of wide-area CL measurement due to quite smaller excitation volume and less surface-sensitive excitation by the *e*-beam, as shown in Fig. 1b–d. Peak-like features were faintly observed at approximately 6.08 eV, which corresponded to the dX_NP_ energy, regardless of the measured positions. However, the peak intensities were comparable to the noise level, making it challenging to engage in precise discussion on the spatial distribution. Nevertheless, a distinguishable 5.5-eV band was found only in the spectrum for the position P6 (red line). In Fig. 3d, the intensity profile of the NBE emission and the 5.5-eV band along the positions labeled P1–P10 in Fig. 3b are displayed. As shown, the emission in the NBE regions was unchanged regardless of the measured positions while the 5.5-eV band emission was spatially distributed with an appearance rate of approximately 10%, which is consistent with the surface coverage rate of approximately 11% of the multilayered aggregates of hBN in the present mBN epilayer.

In conclusion, distinct CL signals were recorded from an mBN epilayer grown on an HOPG substrate by using a home-made CL system capable of large-area and surface-sensitive excitation by an *e*-beam. The CL spectra at 13 K exhibited a predominant 5.5-eV emission band, which has been ascribed to originate from multilayered aggregates of hBN, markedly at thicker areas formed on step edges of the substrate. Conversely, a faint peak at 6.04 ± 0.01 eV was routinely observed from atomically flat areas. Since the energy agreed with the PL peak of 6.05 eV at 10 K that has been assigned as being due to the recombination of phonon-assisted direct excitons of mBN^20^, the CL peak at 6.04 ± 0.01 eV most likely originates from the mBN epilayer. The results encourage to elucidate emission properties of mBN and other low-dimensional materials by using the present surface-sensitive CL system.

## Methods

### Molecular beam epitaxy

An HT-PAMBE system^61^ was used to grow the mBN epilayer on a 10 × 10-mm^2^-area HOPG substrate with a mosaic spread of 0.4°^21–23^. To obtain a fresh graphite surface for the vdW epitaxy, the top surface of the HOPG substrate was removed by exfoliation using an adhesive tape. After the exfoliation, the HOPG substrate was cleaned with toluene to remove any remaining tape residue, followed by an annealing at 200 °C for 4 h in a mixed gas ambient of Ar and H_2_ (5%). To supply a boron flux, a high-temperature effusion cell (Veeco) containing a high-purity (99.999%) natural mixture of ^11^B and ^10^B isotopes was heated up to 1875 °C. To supply an active nitrogen flux, an RF plasma source (Veeco) was used with the power of 550 W and an N_2_ flow rate of 2 sccm. The mBN epilayer was grown at 1390 °C for 3 h. The growth parameters were identical to the mBN epilayers that have been characterized by using OR and PL measurements^20^. Atomic force microscopy measurements^20,28^ revealed that overall surface coverage of the grown BN film was approximately 100%, with approximately 89% of the surface covered predominantly by mBN, together with some small regions of multilayered hBN deposits that nucleated at step edges of the HOPG substrate. No chemical intermixing was observed between the mBN epilayer and the HOPG substrate^21,62^.

## Data Availability

The data that support the findings of this study are available within the article.
